# QHREDGS Enhances Tube Formation, Metabolism and Survival of Endothelial Cells in Collagen-Chitosan Hydrogels

**DOI:** 10.1371/journal.pone.0072956

**Published:** 2013-08-27

**Authors:** Jason W. Miklas, Susan M. Dallabrida, Lewis A. Reis, Nesreen Ismail, Maria Rupnick, Milica Radisic

**Affiliations:** 1 The Institute of Biomaterials and Biomedical Engineering, University of Toronto, Toronto, Ontario, Canada; 2 Center for Vascular Biology Research, Beth Israel Deaconess Medical Center, Boston, Massachusetts, United States of America; 3 Brigham and Women’s Hospital, Cardiovascular Division, Boston, Massachusetts, United States of America (Affiliates of Harvard Medical School, Boston, Massachusetts, United States of America); 4 Department of Chemical Engineering and Applied Chemistry, University of Toronto, Toronto, Ontario, Canada; Biological Research Centre of the Hungarian Academy of Sciences, Hungary

## Abstract

Cell survival in complex, vascularized tissues, has been implicated as a major bottleneck in advancement of therapies based on cardiac tissue engineering. This limitation motivates the search for small, inexpensive molecules that would simultaneously be cardio-protective and vasculogenic. Here, we present peptide sequence QHREDGS, based upon the fibrinogen-like domain of angiopoietin-1, as a prime candidate molecule. We demonstrated previously that QHREDGS improved cardiomyocyte metabolism and mitigated serum starved apoptosis. In this paper we further demonstrate the potency of QHREDGS in its ability to enhance endothelial cell survival, metabolism and tube formation. When endothelial cells were exposed to the soluble form of QHREDGS, improvements in endothelial cell barrier functionality, nitric oxide production and cell metabolism (ATP levels) in serum starved conditions were found. The functionality of the peptide was then examined when conjugated to collagen-chitosan hydrogel, a potential carrier for *in vivo* application. The presence of the peptide in the hydrogel mitigated paclitaxel induced apoptosis of endothelial cells in a dose dependent manner. Furthermore, the peptide modified hydrogels stimulated tube-like structure formation of encapsulated endothelial cells. When integrin αvβ3 or α5β1were antibody blocked during cell encapsulation in peptide modified hydrogels, tube formation was abolished. Therefore, the dual protective nature of the novel peptide QHREDGS may position this peptide as an appealing augmentation for collagen-chitosan hydrogels that could be used for biomaterial delivered cell therapies in the settings of myocardial infarction.

## Introduction

In order to stimulate repair of damaged organs, many research groups have looked towards various cell therapies. However, in the vast majority of cases, injecting cells into a damaged organ/tissue results in a large amount of the cells not remaining at the injury site, while the cells that do remain undergo apoptosis due to lack of nutrients and oxygen [1]. Consequently, novel methods are required to keep cells localized to the injury site and ensure their survival until a vascular network is created [2].

Hydrogels are used as the base biomaterial in many cell therapy strategies as they provide a non-invasive option for cell injection [3]. Since hydrogels can be mixed with cells in liquid state and allowed to gel upon injection, these hydrogel cell suspensions can be administered through a minimally invasive catheter injection procedure. However, the problem of creating a stable vasculature to ensure nutrient and oxygen supply to the injected cells still remains.

To address this, efforts have focused on implanting endothelial cells (EC) within or around biomaterials to help induce vascular formation as well as the use of angiogenic growth factors to help promote vessel formation [4], [5], [6], [7]. The main caveat with using growth factors in a clinical or commercial setting is their high cost and susceptibility to denaturation [8]. Furthermore, it is difficult to quality-control biomaterials modified with growth factors due to the variable shelf-life of growth factors. Covalent or physical immobilization may result in protein inactivation and blocking of the active site. To solve this problem, it is possible to use short peptide sequences that are derived from a specific growth factor to stimulate a similar response in cells. These peptide sequences would be more stable than their growth factor counterparts while, also being less susceptible to conformational changes during binding or encapsulation in biomaterials as they are short linear chains of amino acids. Most importantly, synthetic peptides are significantly more cost effective to produce than recombinant human proteins.

One such peptide sequence of interest for use in biomaterials is the novel angiopoietin-1 (ang1) based peptide QHREDGS [9]. This peptide sequence is based on the fibrinogen-like domain of ang1. Previously, we reported that this peptide sequence was able to promote the survival of neonatal rat cardiomyocytes during paclitaxel (taxol) treatment on glass slides treated with the QHREDGS peptide conjugated to photocrosslinkable chitosan [10]. Our laboratory further characterized this peptide’s function in a collagen-chitosan hydrogel with encapsulated cardiomyocytes finding that the peptide enhanced neonatal rat cardiomyocyte morphology, viability and metabolic activity in a dose dependent manner [11]. The cardio-protective effects of the peptide in concert with the ease with which it can be incorporated into a hydrogel for minimally invasive delivery make this platform appealing as a potential therapy for post myocardial infarction rehabilitation.

Upon myocardial infarction, the ventricular wall loses functional vasculature as well as cardiomyocytes. With time, a non-contractile scar tissue composed of dense extracellular matrix and fibroblasts will be formed. As a result, even if cells were implanted with the aid of a biomaterial for cell localization, the lack of vasculature would result in cell death due to the lack of oxygen and nutrients motivating the need for the development of new biomolecules that will be cardioprotective and enhance blood vessel formation simultaneously. We hypothesised that the QHREDGS peptide would exhibit beneficial effects on endothelial cell survival and tube formation.

Here, we evaluated the effect of soluble QHREDGS peptide on metabolism, permeability and nitric oxide (NO) release of endothelial cell monolayers. Next, the peptide was covalently immobilized to chitosan to create a collagen-chitosan hydrogel, previously shown suitable for myocardial cell injection [11]. Monolayer studies of endothelial cells cultured on the surface of the peptide modified collagen-chitosan hydrogel were performed. These studies allowed us to determine the combined effect of the hydrogel and peptide on endothelial cell phenotype, such as CD31 expression, vascular endothelial cadherin (VE-CAD) expression, cell size and survival. Finally, studies of endothelial cells encapsulated within the peptide modified hydrogel were performed to determine which integrins mediate EC-peptide interactions and to model implantation studies *in vitro*. Collectively our findings suggest that QHREDGS stimulates endothelial cell-cell interactions, enhances cell metabolism and promotes survival.

## Materials and Methods

### Endothelial Cell Maintenance

Dermal human microvascular endothelial cells (HMVEC-D) were obtained (Lonza) and cultured as recommended in the manufacturer’s instructions in full endothelial growth media. Cells were cultured on plates coated with 50 µg/ml collagen I (10 min) (Advanced Biomatrix). Microvascular endothelial cells (MS1 EC) were obtained (ATCC) and cultured as previously described [9]. The human umbilical vein endothelial cells (HUVECs) were received from Lonza (product number C2519A). The cells were then expanded in endothelial cell growth medium-2 (EGM2), which was obtained from Lonza, and frozen down at passage 3. The final concentration of fetal bovine serum in the media was 2%. There was no bovine brain extract and a number of additives were included in the culture media before use as per manufacturer’s instructions. They included: human epidermal growth factor (hEGF), hydrocortisone, GA-1000, vascular endothelial growth factor (VEGF), human basic fibroblast growth factor (hFGF-B), insulin-like growth factor-1 (R^3^-IGF-1), ascorbic acid, and heparin. The media was stored at −20°C and heated to 37°C before use.

### Peptide Modified Chitosan (UP-G113-QHREDGS)

QHREDGS peptide was conjugated to chitosan using 1-ethyl-3-(3-dimethylaminopropyl) carbodiimide HCl (EDC) chemistry in a similar fashion as previously described [10], [11]. To determine the dose response of the peptide, two different concentrations were implemented, a 100 µM and 650 µM concentration of the peptide in proportion to the amount of chitosan present. Chitosan (UP-G113, Novamatrix) was dissolved at 20 mg/ml in 0.9% normal saline and peptide at 10 mg/ml in PBS (Lonza). This solution was then mixed with EDC and *N*-hydroxysulfosuccinimede (S-NHS) dissolved in PBS to a final solution concentration of 5 mg/ml chitosan and the two peptide concentrations: 100 µM for the low peptide (0.5 mg/ml) concentration and 650 µM for the high peptide (3.0 mg/ml) concentration. It should be noted that the ratio of [EDC]/[peptide] and [S-NHS]/[EDC] was kept constant in the two reaction mixtures at 0.8 and 2.75.

### Chitosan-collagen Hydrogel Formulation

The chitosan-collagen hydrogel was created in a similar fashion as previously described [11]. Briefly, the hydrogel had a final concentration of 2.5 mg/ml each of chitosan and collagen; consisted of 203.1 µl pure or peptide-modified chitosan mixed with 100 µl 10X PBS, 681.2 µl of 3.67 mg/ml collagen (BD Biosciences), and 15.7 µl 1N NaOH on ice to make 1 ml of gel solution. The hydrogel was micropipetted onto a tissue culture plate and placed within a humidified incubator at 5% CO_2_ and 37°C to allow the hydrogel to gel.

### Effects of Soluble QHREDGS on Endothelial Cell Monolayers

#### Transwell assay

HMVEC-D were plated on collagen I-coated 12 well Transwell plates (0.4 µm pore size polyester membrane, 6.5 mm diameter inserts) in full EGM2 and grown to confluence N = 3/group with experiments done in triplicate. Some plates were stained using Giemsa to confirm confluence via inverted phase contrast tissue culture microscopy. In wells containing non-stained confluent cells, full media was removed and changed to serum starvation media. Serum starvation media was composed of EGM2 diluted 1 to 5 with endothelial basal media 2 (EBM2) (Lonza). This resulted in a final serum concentration of 1%. Cells were maintained in serum starvation media for 1 hr (37°C, 5% CO_2_). Next, FITC-albumin (Sigma) (1 mg/ml) was added to the top wells (1 hr, 37°C, 5% CO_2_). Linear peptide QHREDGS, scrambled peptide DQSHGER, or matched vehicle control (PBS) was added to a final concentration of 400 µM of peptides (1 hr), human plasma-derived thrombin was added at 2 U/ml (Calbiochem), and cells were incubated (3 hr, 37°C, 5% CO_2_). Fluorescence was quantified using a SpectraMax plate reader (excitation 485 nm/emission 538 nm). The coefficient of albumin permeability (P_a_) was calculated as described [12] and P_a_/Hr determined. The assay was performed at least 4 times with N = 2/group for each repetition.

#### Transendothelial electrical resistance

Collagen I-coated (10 min) ECIS electrode array eight chamber slides were rinsed 5X in Hank’s balanced salt solution (HBSS). HMVEC-D were trypsinized and resuspended in phenol red free EBM (Lonza) plus 1X concentrations of EGM2 (Lonza). Then, ECs were plated and cultured on collagen I-coated chamber slides (37°C, 5% CO_2_) in full growth media in the ECIS 1600R chamber (Applied Biophysics). When ECs were confluent, half of the full media was removed and replaced with phenol red free EBM three times, resulting in a serum starvation media containing 1/6 of the growth factor concentration of full growth media. Linear peptide QHREDGS, scrambled peptide DQSHGER, or matched vehicle control (PBS) to a final concentration of 400 µM peptides was added (1 hr, 37°C, 5% CO_2_). Next, thrombin was added at 2 U/ml and cells incubated up to 3 days (37°C, 5% CO_2_). Transendothelial electrical resistance was monitored throughout the study (ECIS 1600) using N = 2/group with studies conducted in triplicate. The assay was initiated without thrombin for 1 hr to examine the permeability among conditions at baseline. The permeability was assessed for up to 70 hr to demonstrate the peptide’s effect at longer times.

#### Fluorometric nitric oxide assay

The MS1 ECs were cultured in 96-well plates and rinsed twice in phenol red-free DMEM (Invitrogen). Linear QHREDGS peptide, scrambled QHREDGS peptide (DQSHGER), GRGDSP peptide (SynBioSci), angiopoietin-2 (ang2), or ang1 (R&D Systems) or matched vehicles (PBS) were added (N = 6/group with studies done in duplicate) in phenol red-free DMEM/0.5% BSA and incubated (0.5 hr, 37°C, 10% CO_2_). Then, 100 µM L-arginine (Sigma) was added. Where indicated, 100 µM of S-Nitroso-*N*-acetylpenicillamine (SNAP) (Calbiochem) was also added and cells were incubated (1.5 hr, 37°C, 10% CO_2_). A quantitative fluorometric extracellular NO assay (Calbiochem) was performed as per manufacturer’s instructions. Since the final products of NO are nitrates and nitrites, this assay uses nitrate reductase to convert nitrates to nitrites, then uses 2,3-diaminonapthalene and NaOH to convert nitrites to a fluorescent compound. Plates were read using a Wallac 1420 Multilabel Victor^3^ microplate reader (Perkin Elmer), with excitation at 355 nm and emission at 430 nm.

#### Survival and energetics assays

The MS1 ECs were cultured in full media on 96 well plates. Cells were rinsed twice in basal DMEM media (Invitrogen) and linear QHREDGS peptide, scrambled QHREDGS peptide (DQSHGER), or vehicle (PBS) were added in each cell’s basal media plus 0.5% BSA. Cells were incubated for 1 day at 37°C, 10% CO_2_. MTT assays were conducted as described [9]. ATP levels were quantified using the CellTiter-Glo Luminescent Assay (Promega) as per manufacturer’s instructions. NADH/NADPH levels were measured using the CellTiter-96 Aqueous One Assay (Promega). Viability/NAD(P)H levels were used following manufacturer’s instructions. For both assays, N = 6/group, with studies done in duplicate. Luminescence was measured using a Wallac 1420 microplate reader for ATP studies and the optical density (OD) at 490 nm was measured to assess NADH/NADPH levels.

### Effects of QHREDGS Immobilized In Collagen-chitosan Hydrogel on Endothelial Cells

#### Live/dead staining

Live/dead staining was performed on N = 3 gels/group with a HUVEC seeding density of 187,500 cells per hydrogel coated well of a 12 well plate and a working volume of 1 ml during the 4 days of cultivation. The culture media was changed fully every other day. Prior to application of the staining solution, the gels were washed once with 1×PBS (Gibco). The live/dead staining solution (500 µl) consisted of 89% of the total volume being culture media, 10.8% of the final volume being 1.5 µM propidium iodide (Invitrogen), and 0.2% of the final volume being 10 µM 5-(and-6-)-Carboxyfluorescein Diacetate, Succinimid – Mixed Isomers (CFDA-SE) (Invitrogen). After 30 min at 37°C, the staining solution was removed, the gels were washed once with PBS, 200 µl of PBS was added back to the solution and the samples were then observed under a fluorescence microscope (IX81, Olympus). Live and dead cells were counted using the cell counter plug in on ImageJ to determine cell viability.

#### CD31 staining and VE-CAD staining

Immunostaining was performed on N = 3 gels/group with a HUVEC seeding density of 187,500 cells per hydrogel coated well of a 12 well plate and a working volume of 1 ml during the 4 days of cultivation. The cells were fixed in 4% paraformaldehyde for 20 min. The samples were blocked in 10% normal goat serum (NGS) for 40 min at room temperature followed by the application of the primary antibody CD31 (Abcam, 1∶200) or VE-CAD (Abcam, 1∶100) for 18 hr at 4°C. For CD31 staining, the secondary antibody solution consisted of 10% normal goat serum in PBS with 1∶100 anti-mouse antibody Alexa Fluor 488 (AbCam) and 1∶100 DAPI added. For VE-CAD staining the secondary antibody solution consisted of 1.5% normal goat serum in PBS with 1∶100 anti-rabbit antibody Rhodamine (Jackson ImmunoResearch) and 1∶100 DAPI added. The hydrogels were then covered for one hr and incubated at room temperature. The hydrogels were then imaged under a fluorescence microscope (IX81, Olympus). CD31 images were used to determine the average cell number and cell area per condition. Cells were counted using the ImageJ cell counter feature for N = 3 gels/group. For area measurements, cells were traced using the ImageJ free hand selection tool and the diameter was determined using the ImageJ measure feature for N = 3 gels/group.

#### Caspase-3/7 assay

The amount of caspase-3/7 activity after 3 days of culture on the three different hydrogel-peptide concentrations was determined using the Apo-ONE caspase assay (Promega) as per the manufacturer’s protocol. Briefly, 30,000 HUVECs were seeded onto the three different collagen-chitosan hydrogel conditions in a 96 well plate. After 3 days of culture, the cells had one of the three treatments applied in the culture media for 17 hr: DMSO (1∶1000), taxol dissolved in DMSO (2.5 µM) and culture media control (no treatment). Following treatment the caspase-3/7 assay was performed.

#### XTT assay

To normalize the caspase-3/7 activity to cell number, an XTT assay was used to determine the number of cells after three days of culture and 17 hr of treatment in the various hydrogel-peptide groups and treatment conditions. The XTT assay (Biotium) was performed according to the manufacturer’s instructions and as we described previously [11]. A standard curve of known cell numbers was created to correlate the colorimetric data to cell number.

### Encapsulation of Endothelial Cells in Collagen-chitosan QHREDGS Hydrogels

#### Encapsulation time course studies: kinetics and tube-formation

HUVEC encapsulation was performed with the three hydrogel-peptide groups generated as described above. Once the hydrogels had been created, before they were pipetted onto a 96 well plate, they were mixed with a specific number of cells so that 35 µl of gel had 170,000 HUVECs (5·10^6^ cells/ml of gel). Once the hydrogel and cells had been mixed on ice, 35 µl of the hydrogel/cell mixture was pipetted into a 96 well plate. The plate was then placed into the incubator for 30 min (37°C and 5% CO_2_) to allow the hydrogel to gel. After 30 min, 200 µl of culture media was added to each sample and the plate was placed back into the incubator with media changed every 2 days.

To image the HUVECs within the hydrogel, samples were stained using CFDA-SE and imaged at days 2, 4 and 6 as described in the above live/dead staining methods or with CellTracker dye (Invitrogen) and imaged at 6, 18 and 24 hours after seeding. Using CellTracker, encapsulated cells were stained with a 2 µM solution of CellTracker dye in serum free media for 30 min (37°C and 5% CO_2_). The staining solution was then removed and the encapsulated cells were cultured in EGM2 for 30 min (37°C and 5% CO_2_). The media was then removed and the encapsulated cells were washed twice with PBS for 5 min. The encapsulated cells stained with CellTracker dye were then fixed in 4% paraformaldehyde for 15 min and washed in PBS and imaged.

To determine the average longest construct path length, ImageJ was used to draw the longest continuous path along a HUVEC construct without back tracing on the current path. The longest path was determined for all constructs within a single image and then averaged. The average total path length was determined through the addition of all the average longest construct path lengths in an image.

At the end of cultivation of encapsulated cells, the samples were fixed and stained for CD31 as described above. Confocal images were taken using the Olympus FluoView 1000 Laser Scanning Confocal Microscope with FV10-ASW 3.0 Viewer software.

#### Encapsulation integrin blocking assay

HUVEC encapsulation was performed with the three hydrogel-peptide groups generated as described above. The hydrogel solution and the cells were combined so that 35 µl of gel had 170,000 HUVECs (5·10^6^ cells/ml of gel). Once the hydrogel and cells had been mixed on ice, 35 µl of the hydrogel/cell mixture was pipetted into a 96 well plate. The plate was then placed into the incubator for 30 min (37°C and 5% CO_2_) to allow the hydrogel to gel. After 30 min, control Igg antibody cardiac troponin-T (Thermo Scientific) at 6.75 µg/ml, anti-αvβ3 integrin (BD Biosciences) or anti-α5 integrin and anti-β1 integrin (BD Biosciences) were added at 50 µg/ml, concentrations proven effective in previous studies [13], into the culture media and cultured for two days (37°C and 5% CO_2_).

### 1.7 Statistical Analysis

Statistical analysis was performed using SigmaPlot 12.0. Differences between experimental groups were analyzed using one-way and two-way ANOVA depending on the experiment. An α = 0.05 was used for all tests and a normality test (Shapiro-Wilk) and pairwise multiple comparison procedures (Holm-Sidak method) were used for all ANOVA tests. If the equal variance test failed, Kruskal-Wallis One Way Analysis of Variance on Ranks was performed with all pairwise multiple comparisons performed using the Tukey Test. P<0.05 was considered significant for all statistical tests.

## Results

### QHREDGS Decreased Endothelial Cell Permeability upon Thrombin Treatment

**Figure 1:**
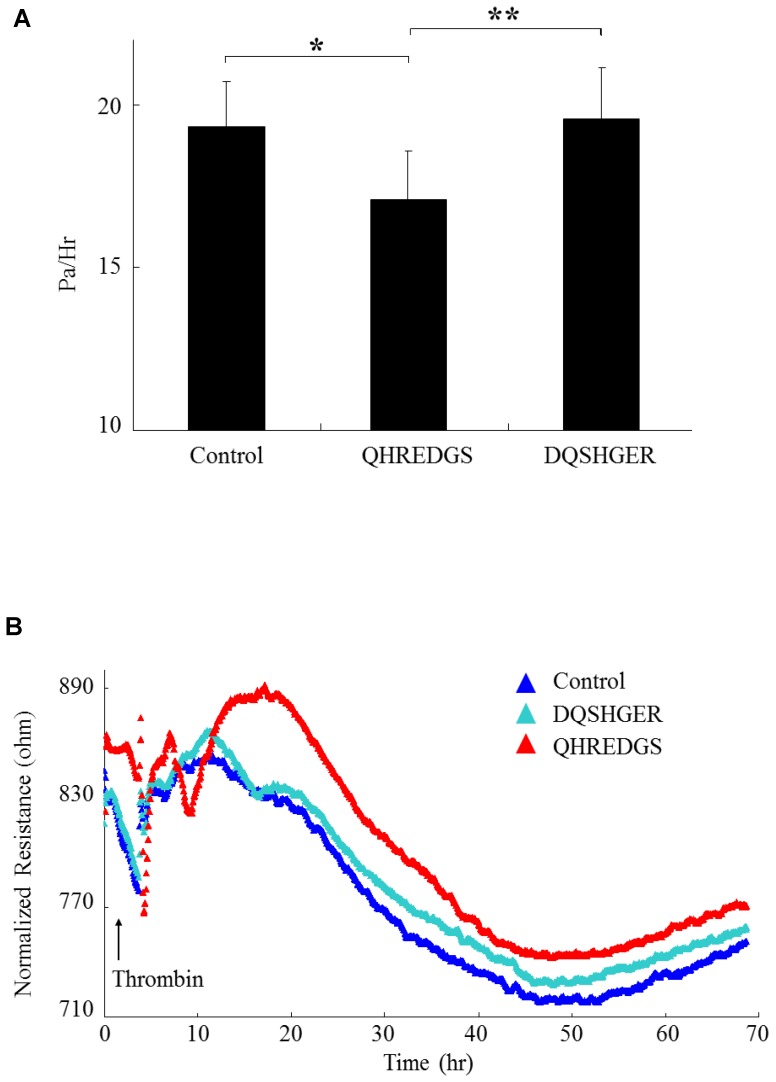
Linear QHREDGS Peptide Decreases Endothelial Cell Permeability. (A) HMVEC-D were cultured on collagen I and permeability was assessed using a Transwell assay (N = 3/group). Endothelial cells were serum starved (1 hr) and FITC labeled albumin was added to the top well and cells were incubated. QHREDGS or scrambled (DQSHER) peptides at 400 µM or control (PBS) were added (1 hr). Thrombin was added to induce permeability and the amount of FITC-linked albumin in the bottom well was quantified (Pa/hr) (3 hr). Peptide QHREDGS reduced HMVEC-D permeability (*P = 0.004) compared to control and scrambled peptide (**P = 0.001) groups. (B) Transendothelial electrical resistance was measured in HMVEC-D cultured on collagen I. HMVEC-D were serum starved and QHREDGS or scrambled (DQSHER) peptides at 400 µM or control (PBS) were added (1 hr). Thrombin was added to reduce resistance and resistance was measured as a function of time (N = 2/group). Peptide QHREDGS increased QHREDGS resistance compared to control and scrambled peptides. Red coloured triangles indicate normalized resistance values for the QHREDGS treated group.

The effect that the peptide had on the permeability of HMVEC-D was determined using a transwell assay. **Figure 1A** shows that the peptide treated HMVEC-D significantly decreased (P<0.004) the permeability of the confluent cell layer in comparison to the control and the scrambled peptide group. Next, the effect of the peptide on endothelial barrier function was evaluated at baseline and in response to thrombin using a transendothelial electrical resistance (TEER) assay, **Figure 1B**. At time 0, all three groups had similar resistance. However, in the group treated with the peptide QHREDGS, resistance rapidly increased. At 1 hr, thrombin was added to induce endothelial cell permeability and disrupt barrier function. Thrombin, which disrupts endothelial cell barrier function, was added to each of the three conditions of HMVEC-D culture and, resulted in a decrease in resistance of the endothelial layer of all three conditions. However, the resistance of the QHREDGS peptide treated group recovered to a greater extent and remained at a higher resistance level compared to the scrambled and control treated HMVEC-D groups throughout the 70 hr study. Peptide QHREDGS increased HMVEC-D barrier function at baseline and with thrombin. Thus, similar to ang1 [14], [15], peptide QHREDGS attenuated EC permeability and improved intercellular barrier integrity.

### QHREDGS Enhanced Endothelial Cell NO Production and Metabolism

**Figure 2:**
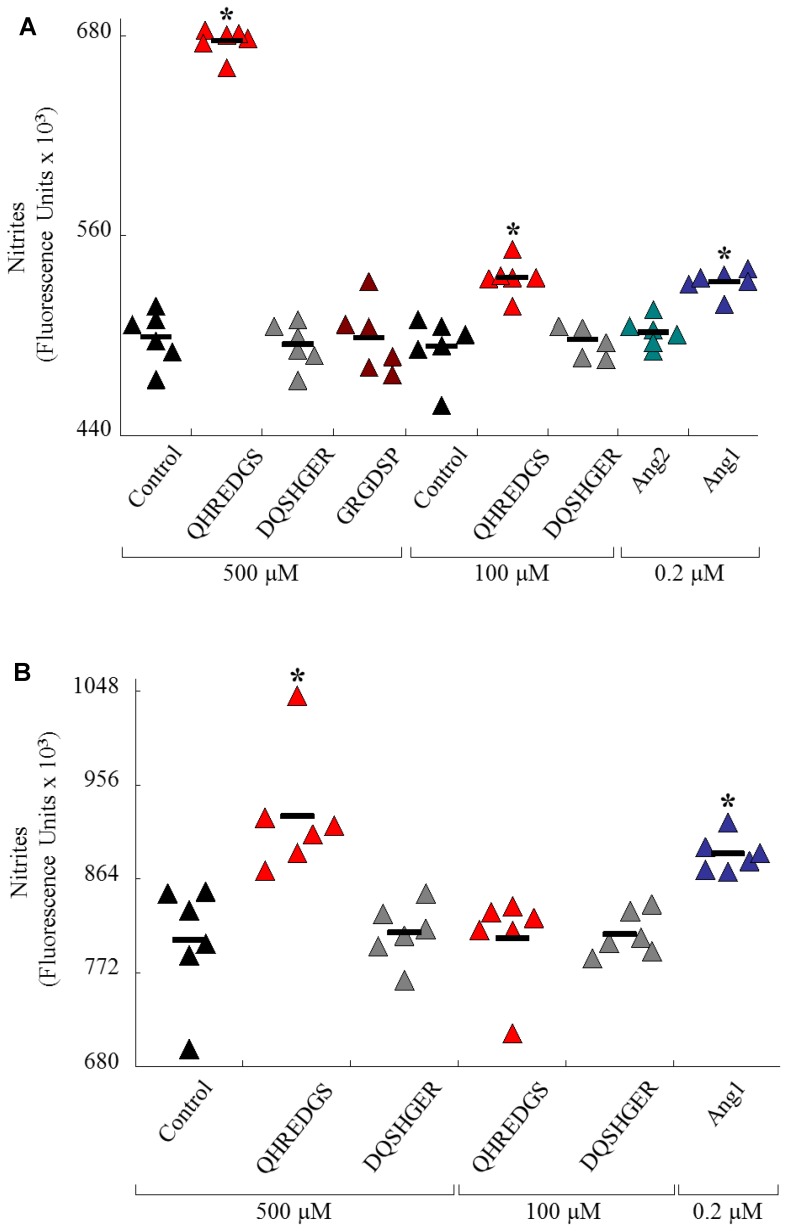
Linear QHREDGS Peptide Increases Nitric Oxide Release from Endothelial Cells. (A) Mouse MS1 endothelial cells were incubated with linear QHREDGS peptide, scrambled QHREDGS peptide (DQSHGER), GRGDSP peptide, control (PBS), or 200 nM angiopoietin-2 (ang2) or angiopoietin-1 (ang1) (0.5 hr). Then, 100 µM L-arginine was added and cells incubated (1.5 hr). Extracellular nitric oxide (NO) levels were measured (N = 6/group). In graphs, the mean value is indicated with a bar. Linear QHREDGS peptide increased NO levels at 500 and 100 µM (*P≤0.00001) and ang1 also increased NO levels (**P = 0.0002). Ang2, GRGDSP, and scrambled QHREDGS peptides had no effect on NO levels compared to controls. (B) MS1 endothelial cells were incubated with peptides and proteins as above, except SNAP (NO donor) was added to the 1.5 hr incubation with L-arginine. In graphs, the mean value is indicated with a bar. Linear QHREDGS peptide at 500 µM and ang1 increased NO levels (*P<0.002). Scrambled QHREDGS peptide and 100 µM QHREDGS did not affect NO levels. Triangle color code: control (black), scrambled peptide (gray), QHREDGS peptide (red), GRGDSP peptide (burgundy), ang2 (green) and ang1 (blue).

The addition of the peptide to MS1 endothelial cell cultures increased the release of nitric oxide in a dose dependent manner (**Figure 2A**). Examining the effect ang1 had on nitric oxide release, a statistically significant increase in comparison to the PBS control was found at an ang1 concentration of 200 nM. The control, RGD-based peptide sequence GRGDSP as well as ang2 did not show a statistically significant increase in nitric oxide release in comparison to the PBS controls. Next, the peptide QHREDGS, which is derived from ang1, was tested at two different concentrations, 100 µM and 500 µM. Both concentrations showed a statistically significant increase in nitric oxide production in comparison to their respective PBS controls and there was a dose response found with the 500 µM QHREDGS peptide inducing a greater amount of nitric oxide produced compared to the 100 µM condition. **Figure 2B** shows that when MS1 endothelial cells were pre-treated with NO donor S-Nitroso-N-acetylpenicillamine, the cells treated with either 200 nM of ang1 or 500 µM of QHREDGS peptide released a significantly greater amount of nitric oxide compared to their respective PBS controls. No statistically significant difference was found between the scrambled peptide group or the 100 µM QHREDGS and their respective PBS controls.

**Figure 3:**
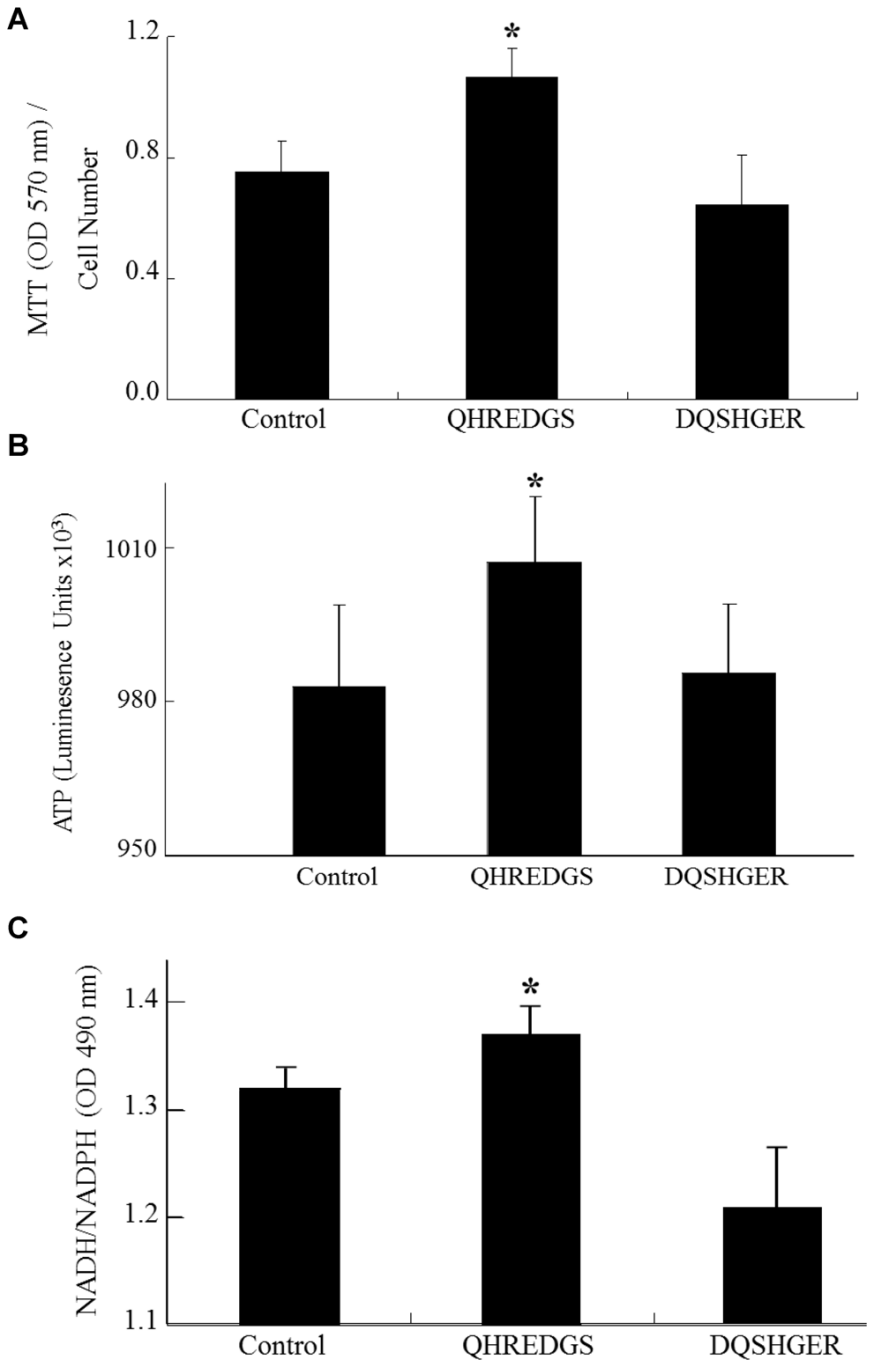
Linear QHREDGS Peptide Increases Endothelial Cell Survival and Energetics. (A-B) MS1 endothelial cells were incubated with 500 µM QHREDGS or scrambled QHREDGS peptide (DQSHER) or control (PBS) overnight. (A) Cells were incubated for 4 hr with MTT, absorbances measured at 570 nm, and values normalized to cell number (N = 5/group). Peptide QHREDGS increased MS1 endothelial cell viability (*P = 0.001) as indicated by the increased processing of MTT by mitochondria. (B) QHREDGS peptide increased endothelial cell ATP levels, whereas scrambled QHREDGS had no such effect (*P = 0.03). (C) MS1 endothelial cells were incubated with 500 µM QHREDGS or scrambled QHREDGS peptide or control (PBS) for 3 hr. QHREDGS peptide increased endothelial cell NADH/NADPH levels (*P = 0.05) suggesting increased cellular energetics. Optical density (OD).

The effect that the peptide had on MS1 endothelial cell survival and metabolic activity in serum-starved conditions is shown in **Figure 3** through the measurements of MTT, ATP and NAD(P)H levels. **Figure 3A** shows that in serum-starved conditions, the peptide group had a significantly higher (P = 0.001) amount of MTT being reduced, meaning more cells were viable and active, in comparison to PBS controls. The scrambled peptide group, DQSHGER, had similar MTT levels as the PBS control. Additionally, in serum-starved conditions, the QHREDGS peptide group had a significantly higher (P≤0.03) ATP level in comparison to the PBS control (**Figure 3B**). The scrambled peptide group had a similar ATP level to the PBS control. The QHREDGS peptide also significantly increased the NAD(P)H levels in MS1 ECs in serum starved conditions compared to the PBS control and scrambled peptide (P = 0.001; **Figure 3C**).

### Cultivation of HUVECs on QHREDGS Modified Collagen-Chitosan Hydrogels Led to Improved Cell Viability

**Figure 4:**
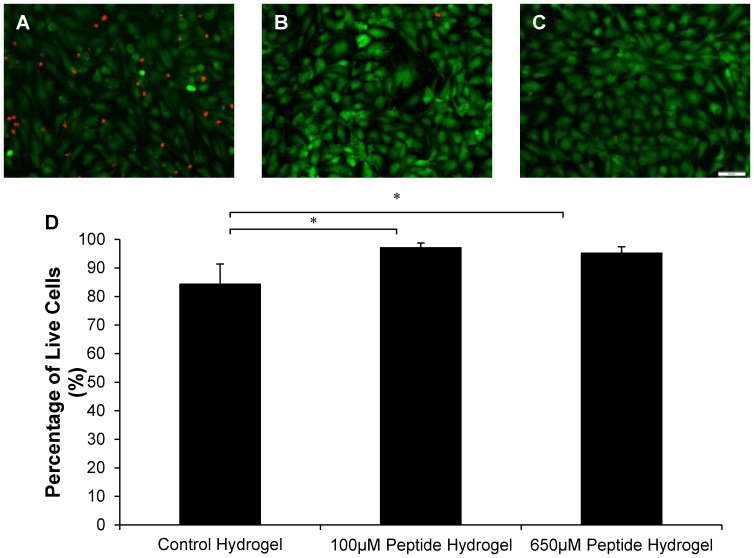
Cultivation of HUVECs on QHREDGS Modified Collagen-Chitosan Hydrogel Leads to Improved Viability. Characterization of HUVECs on collagen-chitosan hydrogels with different peptide concentrations after 4 days of cultivation. 187,500 cells were seeded on the freshly made hydrogels. (A) Control hydrogel, (B) 100 µM peptide hydrogel and (C) 650 µM peptide hydrogel with HUVECs stained for live/dead imaging with CFDA-SE for live cells (green) and PI for dead cells (red). (D) Percentage of live cells. All images are 20X magnification with scale bar of 50 µm. *P<0.05.

HUVECs cultured on the QHREDGS modified collagen-chitosan hydrogels for 4 days had 84% viability for the control hydrogel and 97% and 95% for the 100 µM and 650 µM peptide modified hydrogel conditions (**Figure 4A, C**). Using One-Way ANOVA, a statistically significant difference between the control hydrogel and the 100 µM and 650 µM peptide modified hydrogel conditions were found (P = 0.036 and P = 0.046).

**Figure 5:**
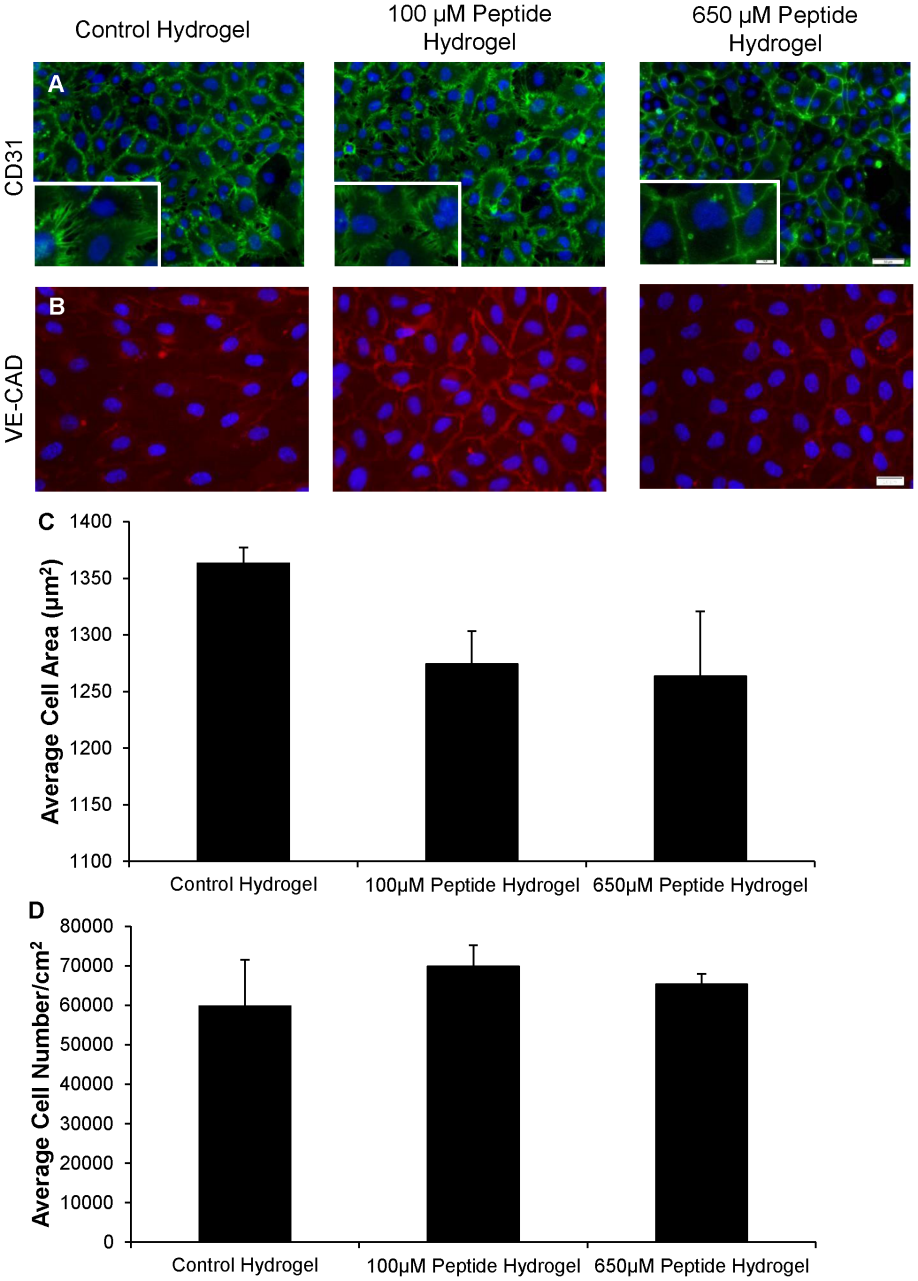
QHREDGS Modified Collagen-Chitosan Hydrogels Support Expression of CD31 and VE-CAD in Endothelial Cells Characterization of HUVECs on collagen-chitosan hydrogels with different peptide concentrations after 4 days of cultivation. 187,500 cells were seeded on the freshly made hydrogels. (A) HUVECs stained for CD31 (green) and cell nucleus (blue) in control, 100 µM peptide and 650 µM peptide hydrogels. 20X magnification with scale bar of 50 µm and higher magnification inset scale bar of 10 µm. (B) HUVECs stained for VE-CAD (red) and cell nucleus (blue) stained in control, 100 µM peptide, and 650 µM peptide hydrogels. 40X magnification with scale bar of 20 µm. (D) Average cell area. (E) Average cell number per cm^2^.

CD31 is an integral membrane protein that mediates cell-to-cell adhesion. CD31 is expressed constitutively on the surface of adult and embryonic endothelial cells and mediates endothelial cell-cell interactions and angiogenesis. CD31 staining for HUVECs cultivated as monolayers on top of the collagen-chitosan hydrogels, shown in **Figure 5A**, was positive for all three of the hydrogel groups. There was a closer association of HUVEC membranes in the 650 µM peptide hydrogel group, while the 100 µM peptide hydrogel and control hydrogel groups showed HUVECs intercellular connections were not as tight. Furthermore, the cells cultivated on the 100 µM and 650 µM hydrogel had more pronounced staining for VE-CAD compared to those cultivated on the control hydrogel (**Figure 5B**). Cell area for HUVECs on each of the three hydrogel groups, shown in **Figure 5C**, were found to be comparable at 1360, 1270 and 1260 µm^2^ for the control, 100 µM and 650 µM peptide hydrogel groups. The average cell density (**Figure 5D**) in each of the three hydrogel groups was also found to be similar with values of 60,000, 70,000 and 65,000 cells/cm^2^ for the control, 100 µM and 650 µM peptide hydrogel groups. These data are consistent with known actions of ang1. Ang1 does not promote EC proliferation [16] and as such, QHREDGS was not expected to increase EC density or cell area. However, similar to ang1 [17], peptide QHREDGS did increase fortification of intercellular connections, as evidenced by the change in CD31 and VE-CAD staining showing less space between cells and more cell to cell connections.

### QHREDGS Promotes Endothelial Cell Survival

**Figure 6:**
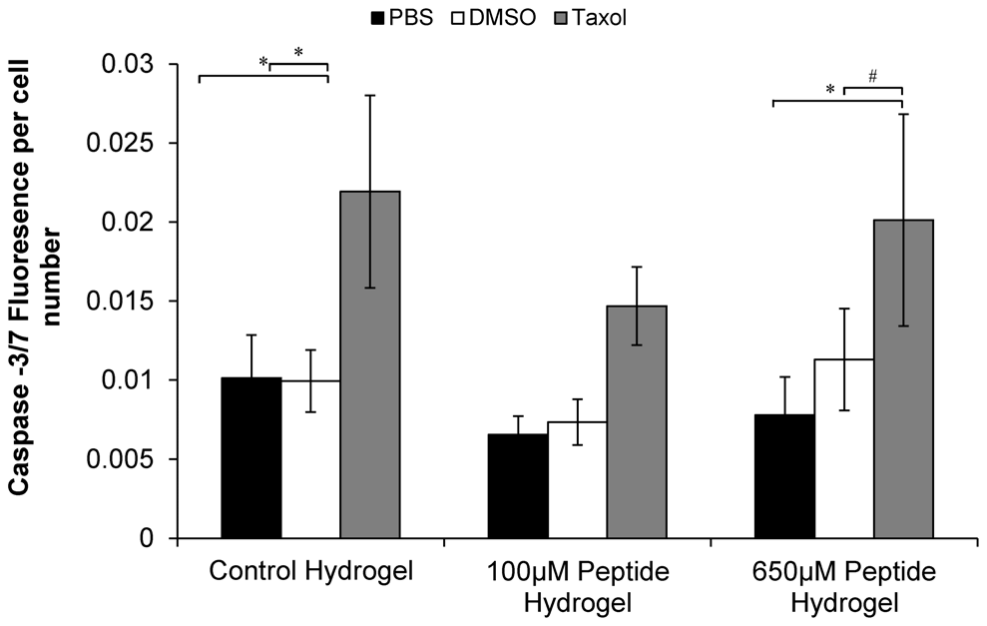
QHREDGS Provides a Protective Effect on Endothelial Cells Undergoing Apoptosis on Collagen-Chitosan Hydrogels. Caspase-3/7 activity, normalized to cell number, is shown for each of the three hydrogel-peptide concentrations. After three days of HUVEC cultivation, each hydrogel group was treated three different ways for 15 hr. The treatments were: PBS control, DMSO carrier and Taxol 2.5 µM. * P<0.05, # P = 0.051.

Upon treatment with paclitaxel (taxol), capsase-3/7 activation was significantly increased in cells cultivated on control and peptide modified collagen-chitosan hydrogels with 650 µM QHREDGS peptide in comparison to their respective PBS control as well as their DMSO carrier alone (**Figure 6**). Interestingly, when cells were cultivated on the peptide modified hydrogels with 100 µM QHREDGS, there was no statistically significant increase in caspase-3/7 activation between the taxol, DMSO and PBS conditions.

### Lower Concentrations of QHREDGS Led to more Robust Tube-Like Structure Formation by Encapsulated Endothelial Cells

**Figure 7:**
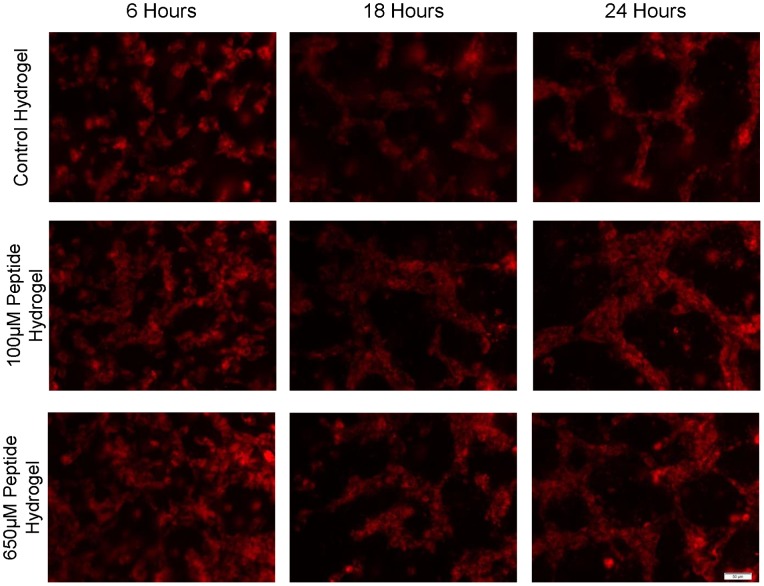
QHREDGS Modified Collagen-Chitosan Hydrogels Expedite Kinetics of Endothelial Cell Tube Formation. Encapsulation of HUVECs within the control, 100 µM peptide and 650 µM peptide hydrogel groups with HUVEC membranes stained with CellTracker dye (red). Images taken at 6, 18, and 24 hr to show the progression of HUVEC constructs forming within the hydrogel. All images were taken at 20X magnification. Scale bar is 50 µm.

**Figure 8:**
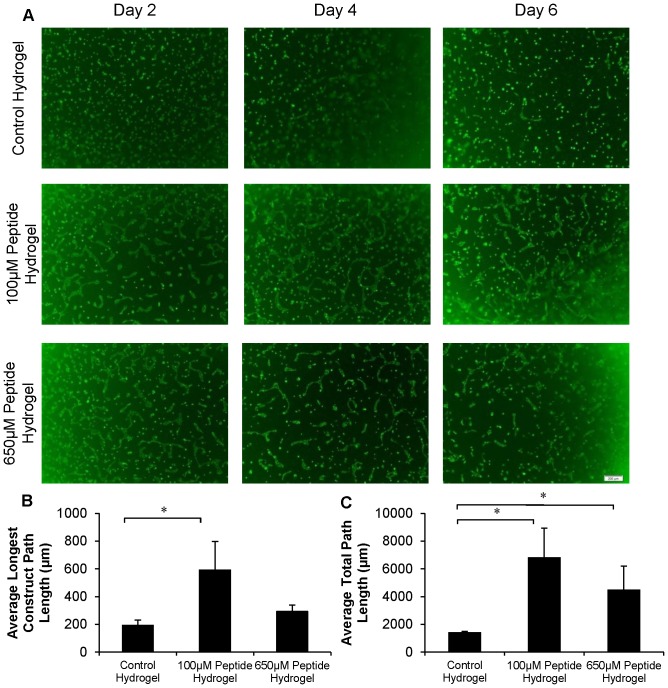
QHREDGS Modified Collagen-Chitosan Hydrogels Enhance Tube-Like Structure Formation of Encapsulated HUVECs. Encapsulation of HUVECs within the control hydrogel and two peptide modified hydrogel groups. (A) HUVECs stained with CFDA-SE for live cells (green). Images taken every other day to show the progression of HUVEC constructs forming within the hydrogel. (B) Average longest construct path length determined on day 4. (C) Average total path length of constructs determined on day 4. * P<0.05. All images are taken at 4X magnification. Scale bar is 200 µm.

Upon encapsulation of HUVECs in QHREDGS modified collagen-chitosan hydrogels, changes in tube-formation kinetics, morphology, and organization were observed. We have performed early stage vascular morphogenesis studies to better understand the peptide’s effect on promoting EC tube-like structures (**Figure 7**). The 18 and 24 hr time points for the peptide groups exhibited more robust tube-like formations with the 100 µM peptide group having the densest networks of tube-like structures that were wider compared to those in the control or 650 µM conditions (**Figure 7**). Over the course of 6 days, the HUVECs found within the control hydrogel continued to show minor changes in cell morphology and organization (**Figure 8**). The majority of the cells remained rounded throughout the time course. In the 100 µM peptide hydrogel group, the largest degree of change in cell structure and morphology was observed. By day 2, many of the HUVECs had clustered together to form elongated tube-like structures within the hydrogel with the peak of this phenomenon occurring on day 4. By day 6, these tube-like structures began to regress in both number and size. The 650 µM peptide hydrogel displayed a faster and less pronounced effect than the 100 µM peptide hydrogel group, with peak construct size and elongation found on day 2 and regression beginning to occur on day 4. Some HUVEC tubular structures remained at day 6 but were smaller in comparison to those observed on day 2.

Quantification of the tube-like constructs was then performed on day 4 hydrogels. **Figure 8B** shows the average longest single path that can be drawn, without overlapping paths, on a HUVEC tube-like structure. This result showed that the peptide hydrogel groups had on average longer tube constructs in comparison to the control hydrogel group with, the 100 µM peptide hydrogel group having the largest average constructs of the three hydrogel groups. A statistically significant difference in the longest path length was found between the 100 µM peptide modified hydrogel and the control hydrogel (P<0.05). Quantification of the size and frequency of HUVEC constructs formed was performed by the addition of all the total path lengths for a single image which was then averaged across multiple images to give an average total path length per image. The results, **Figure 8C**, showed that the peptide modified hydrogels had a higher average total path length, or in other words a higher abundance of HUVEC constructs, in comparison to the control hydrogel group with the 100 µM peptide hydrogel group having the highest average total path length of the three groups. A statistically significant difference in the total path length was found between the 100 µM peptide hydrogel and the control hydrogel (P<0.05) as well as the 650 µM peptide hydrogel and control hydrogel (P<0.05).

**Figure 9:**
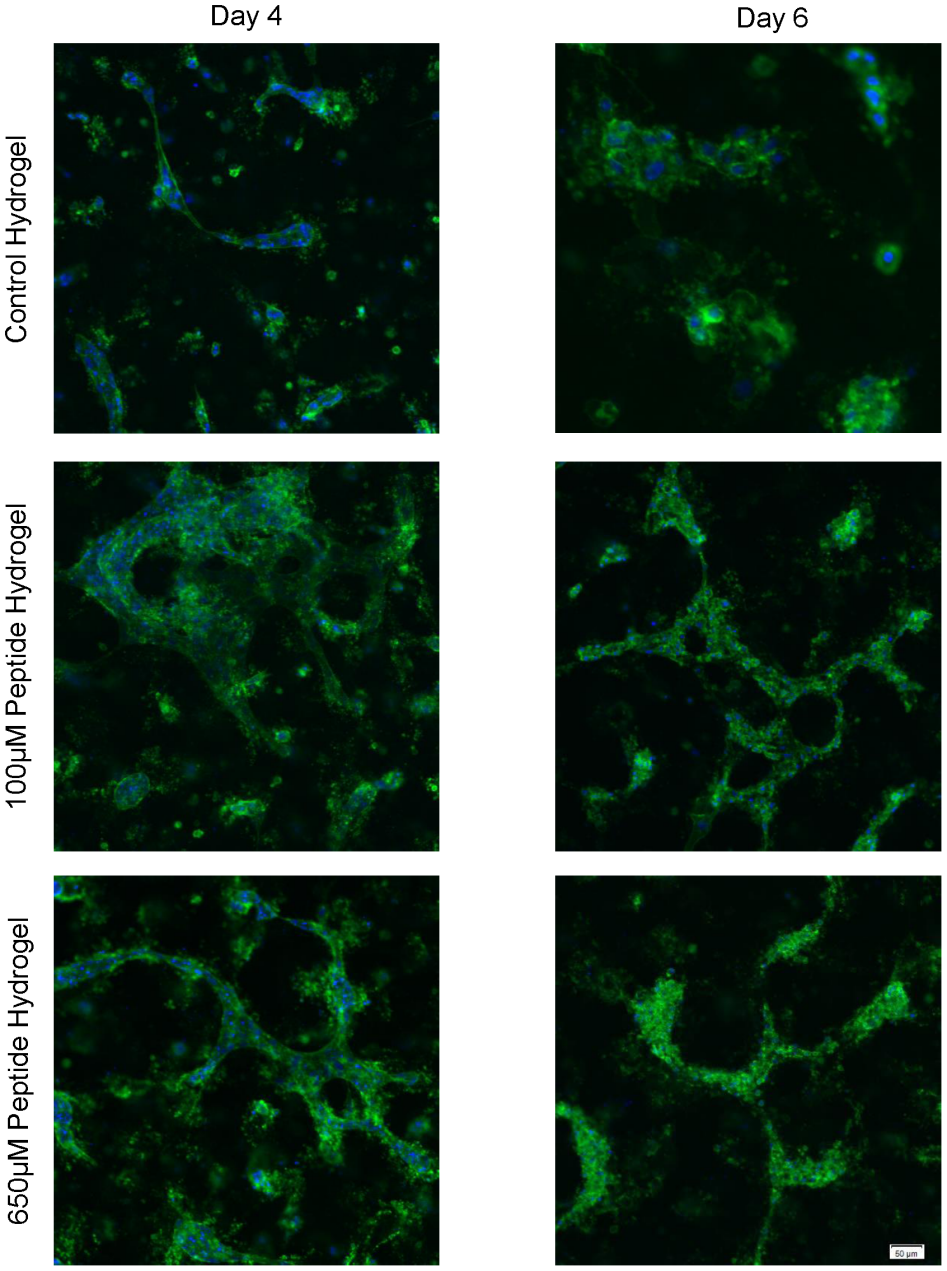
CD31 Staining of Tube-Like Structures is Enhanced in QHREDGS Peptide Modified Hydrogels. Confocal microscopy of encapsulated HUVECs within the control hydrogel and two peptide modified hydrogels after 4 and 6 days of cultivation. CD31 (green) and cell nucleus (blue). All images were taken at 20X magnification. Scale bar is 50 µm.

Confocal images of CD31 stained cells helped to better elucidate the structure of the HUVEC tube constructs seen in the encapsulation experiments (**Figure 9**). As was previously stated, the peptide hydrogels had larger constructs in comparison to the control hydrogel group. The control hydrogel constructs were comprised of few cells with minimal cell elongation and organization in both the day 4 and 6 images. In contrast, the 100 µM peptide hydrogel group had extensive tubes on day 4, consisting of many elongated and connected cells staining strongly for CD31. On day 6, the tube constructs began to regress even in the 100 µM group. The 650 µM peptide group mirrored the 100 µM peptide hydrogel group, however, in general the construct size and cell elongation at days 4 and 6 were smaller in the 650 µM peptide group compared to the control.

### Integrins αvβ3 and α5β1 Mediate Tube Formation by HUVECs in Peptide Modified Hydrogels

**Figure 10:**
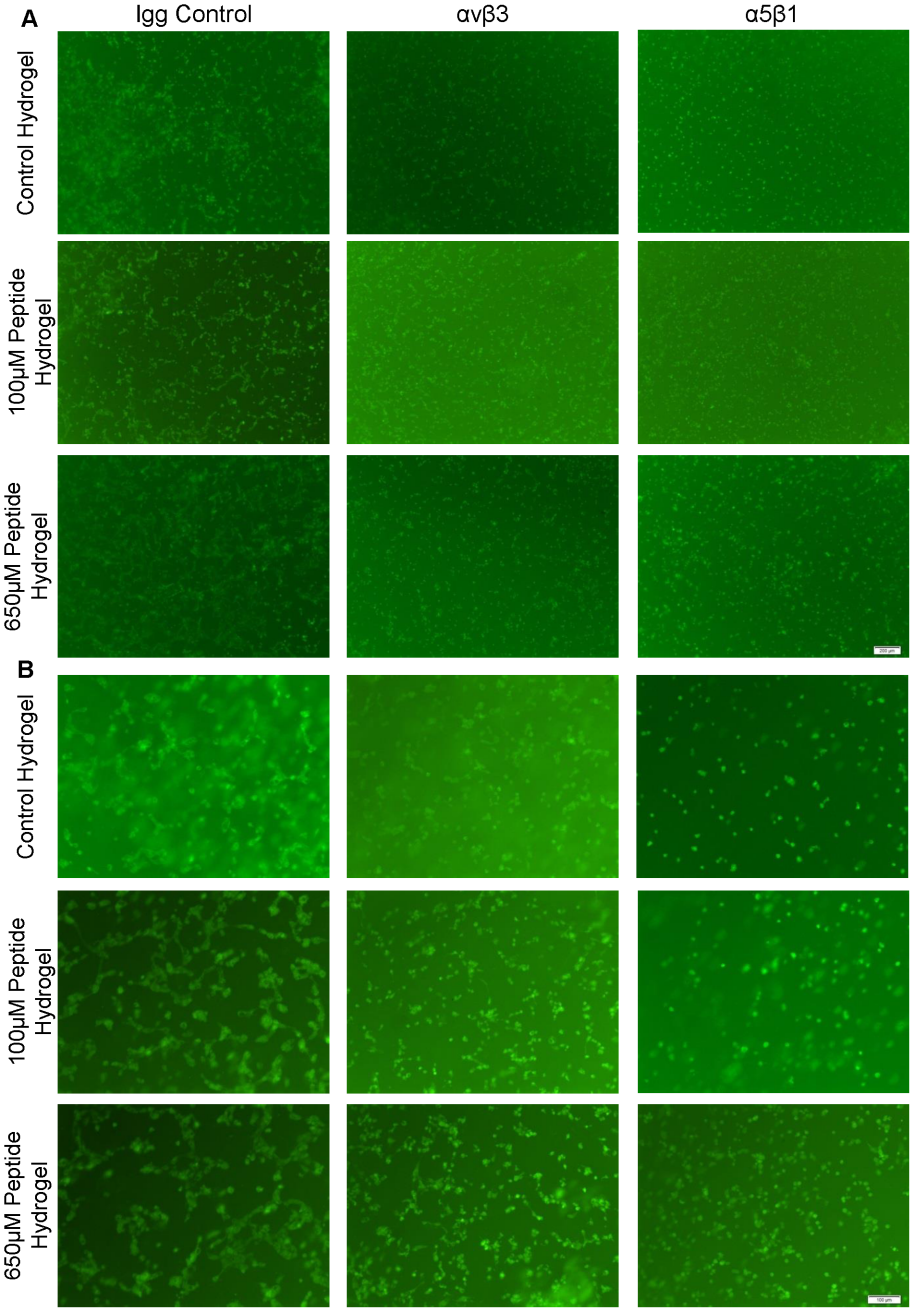
Blocking αvβ3 or α5β1 Integrin Receptor Inhibits Formation of QHREDGS Tube-Like Structures by Encapsulated HUVECs. Encapsulation of HUVECs within the control hydrogel and two peptide modified hydrogel groups for two days in the presence of either an Igg control, αvβ3 or α5β1 antibody. HUVECs stained with CFDA**-**SE for live cells (green). Images taken at the end of the two day cultivation to examine tube-like structure formation. (A) Images taken at 4X magnification. Scale bar is 200 µm. (B) Images taken at 10X magnification. Scale bar is 100 µm.

When the integrin receptor αvβ3 was blocked, the ability of HUVECs to form robust tube-like structures in the peptide modified hydrogels was hindered (**Figure 10**). When integrin receptor α5β1 was blocked in the cells encapsulated in the peptide modified hydrogels, HUVECs did not form tube-like structures, elongate or cluster and instead remained as round isolated cells. The cells in the control hydrogel with blocked αvβ3 integrin, exhibited a similar appearance as those cultivated with the control Igg antibody, while α5β1 integrin blocking completely abolished tube formation in the control hydrogel. Since integrin receptor αvβ3 is specific to fibrin we did not expect a significant effect of its blocking on the HUVECs ability to interact with the control hydrogel which, is comprised of only collagen and chitosan. α5β1 is the shared integrin receptor for both collagen and fibrin, thus the use of α5β1 integrin antibody prevented HUVEC’s interaction with the major components of the hydrogel (collagen and QHREDGS peptide), while blocking of αvβ3 integrin most likely prevented HUVECs from interacting with the peptide only.

## Discussion

Modified biomaterials are required to provide protection for cells introduced into the hostile environment of a wounded tissue. Consequently, many groups are focusing on discovering novel peptides, derived from growth factors, to enhance the properties of biomaterials and protect cell activity during the initial stage of implantation. Presented in this paper is the use of a novel peptide, QHREDGS, based on the fibrinogen-like domain of angiopoietin-1, to enhance endothelial cell survival and preserve endothelial cell functionality. We examined the effects of the soluble peptide on endothelial cells cultured on tissue culture plastic as well as the peptide covalently immobilized in a hydrogel suitable for myocardial cell injection [11]. We demonstrated previously that QHREDGS promoted survival and functional properties of cardiomyocytes cultured in an *in vitro* collagen-chitosan hydrogel [11]. The current study revealed that the peptide QHREDGS had a dual protective effect for both cardiomyocytes and endothelial cells. QHREDGS is thought to signal both cardiomyocytes and endothelial cells through integrin binding domains since the peptide is based on the fibrinogen-like domain of ang1. The cells are thought to bind to QHREDGS via the α_V_β_3_ and/or α_5_β_1_ integrins [9], [18], due to the homologous nature of the fibrinogen-like domains of angptl3, ang1 and ang2 [9], [19]. The dual protective nature of QHREDGS positions the peptide as a beneficial modifier of biomaterials for myocardial cell therapy.

Peptide sequences are being used in favour of growth factors, for the modification of biomaterials, due to the plethora of issues surrounding the use of growth factors. These include: a) undesirable immune responses as the growth factors are often isolated from non-human organisms, b) the proteins are subject to proteolytic degradation, with further degradation occurring during an inflammatory response, and c) their binding to biomaterials could block the domain of interest making the growth factor ineffective [20]. Specifically for ang1, the production of this recombinant protein has been hindered by its insolubility and the variability of the protein’s activity [21]. One peptide sequence of much interest has been the cell binding motif Arg-Gly-Asp (RGD) [22]. RGD has been used in many biomaterials to improve endothelial cell adhesion, proliferation and migration [20], [23], [24], [25]. While the peptide that we present is thought to also interact with endothelial cells through integrin binding domains, QHREDGS also has the added functionality in its ability to mitigate cell apoptosis.

Biomaterials implanted into patients need to ensure survival of the encapsulated cells and promote the formation of a stable vasculature with the host in order for cells suspended within the biomaterial to survive implantation in the long term. We have shown previously that the peptide QHREDGS can be effectively conjugated to chitosan to create a peptide modified collagen-chitosan hydrogel [11]. The prior study by Rask *et al.* used a photocrosslinkable Az-chitosan with the peptide QHREDGS bound to chitosan. The material was spun coated and UV-crosslinked onto glass cover slips [10]. The reason behind moving away from the photocrosslinkable Az-chitosan to a collagen-chitosan hydrogel was that we wanted to create a material that could be easily injected into the heart and gel *in situ* rather than requiring UV crosslinking, to avoid limitations of UV penetration depth. The new *in situ* gelable formulation was demonstrated to be suitable for culture of encapsulated heart cells and injection into infarcted hearts [11].

Two other available alternatives to recombinant ang1 are COMP-Ang1, which is a modified ang1 recombinant protein containing a minimal coiled-coil domain [21], and Vasculotide, which is an ang-based peptide-mimetic compound [26]. COMP-Ang1 has been shown to improve endothelial cell barrier function, mitigate radiation induced endothelial cell apoptosis and induce the postcapillary venule and venous ends of capillaries to enlarge due to endothelial cell proliferation leading to enhanced blood flow [21], [27], [28]. However, COMP-Ang1 is still a recombinant protein that could lead to a foreign body immune response. Vasculotide has been shown to induce cell migration, MMP2 release, and protection from serum withdrawal-induced apoptosis in endothelial cells [26]. Furthermore, Van Slyke *et al.* found that Vasculotide induced neovascularization *in vivo*, but its effect on cardiomyocytes has not been explored. QHREDGS peptide could be a more appealing modifier for cardiac cell therapy biomaterials due to its dual effect of improving both cardiomyocyte [11] and endothelial cell functionality.

Ang1 has been shown to have a variety of influences on EC migration, permeability, inflammation and vascular maturation [29] activating a number of signalling cascades through Tie2 receptor. Splitting ECs into two broad categories: 1) quiescent ECs that are in contact with one another or 2) motile non-contacting ECs; differences in the Tie2 receptor location on the cell were discovered and lead to a greater understanding of ang1 situation specific signalling [30], [31]. In confluent ECs, ang1 induces Tie2 translocation to cell-cell contacts which results in the formation of homotypic Tie2-Tie2 trans-associated complexes [31], [32]. These complexes then preferentially activate the AKT pathway resulting in increased survival signals [33], increased endothelial NO synthases (eNOS) synthesis [29] and reduced permeability through the sequestration of Src by mDia which reduces the internalization of VE-CAD [34]. When motile and non-contacting ECs are stimulated by ang1 the Tie2 receptor is instead located preferentially at cell-substratum contacts which facilitates ECM bound ang1 to trigger Dok-R, ERK1/2 and MAPK signalling [30], [31]. When ECs are stimulated by ang1 in this context, cell migration is stimulated. Cascone *et al.* also demonstrated that there was an association between Tie2 and the integrin α5β1 on ECs and that this relationship was regulated through the engagement of α5β1 to fibronectin [18]. The activation of α5β1 resulted in Tie2 phosphorylating at low ang1 concentrations yet, attenuated activation at high ang1 concentrations. This combined α5β1-Tie2 activation at a low concentration of ang1 resulted in promoting EC survival and inducing persistent EC motility [18].

Preservation of endothelial cell barrier functional integrity, protection against vascular leakiness investigated in microvascular endothelial cells, and overall, improved endothelial cell health, survival, and energetics are all characteristics associated with peptide QHREDGS (**Figure 1** **–** **3**). Thus, QHREDGS has overlapping functional effects on cardiomyocytes as shown in our previous studies [10], [11] and endothelial cells, as shown here.

NO is a short-lived molecular messenger formed from L-arginine by NO synthases (NOS). NO made by endothelial NOS in particular is vital to EC function and protects against hypertension and cardiovascular injury [35], [36]. It is a potent vasodilator and inhibits smooth muscle cell (SMC) proliferation and migration, and matrix turnover [37]. NO is therefore instrumental in both acute and chronic regulation of vascular tone. Ang1 enhances NO production through Tie2 phosphorylation which leads to the phosphorylation of the p85 subunit of phosphoinositide 3-kinase (PI3K), resulting in the activation of AKT [33], [38]. AKT has been shown to promote endothelial nitric oxide synthase production [29].

By closely observing the y-axis scale in **Figures 2A and B**, it is possible to see that the levels of NO were lower in all groups at baseline condition (**Figure 2A**) compared to the levels in the presence of SNAP, the NO donor (**Figure 2B**). QHREDGS peptide and ang1, exhibited enhanced NO production upon addition of L-arginine, compared to the control groups. In the presence of a competing source for NO generation, namely SNAP, the effects of QHREDGS peptide were dampened, as expected. However, without SNAP-induced NO contributions, the effect of QHREDGS peptide and ang1 alone were elaborated. The RGD peptide had no effect on nitric oxide release at 500 µM (**Figure 2A**). It also had no effect on cardiomyocyte survival at any of the concentrations we tested in previous work [10], [39], thus we concluded it was not necessary to systematically use this control in all of our experiments. It is possible that a greater effect of the QHREDGS peptide on NO release could be gained by using an even higher concentration of the QHREDGS peptide, however, these higher concentrations begin to move out of physiologically relevant doses. We believe that the concentration ranges presented provide the necessary information about the peptide’s ability to instigate the release of nitric oxide and these also fall into the ranges we investigated previously for their effects on cardiomyocytes [11].

Ang1 has been shown to improve skin cell metabolism and viability [40]. Consistent with this functional role, we showed that ang1-derived peptide QHREDGS also promoted cell viability and metabolism in ECs and in cardiomyocytes [9], [10]. Furthermore, ang1 did not promote EC proliferation, but it did increase EC tube formation and survival, promoted tight junction formation and reduced EC permeability [41]. Studies of both immobilized and soluble peptide QHREDGS showed that this peptide reduced EC permeability, increased electrical resistance, and fortified endothelial cell-cell connections. Thus, the peptide QHREDGS has the potential to prevent vascular leakage in pathologic settings wherein increases in vascular permeability are problematic (e.g. sepsis, tumor angiogenesis, proliferative diabetic retinopathy, age-related macular degeneration, blood brain barrier breakdown).

The rationale for using 100 µM and 650 µM concentrations of the peptide was based on previously reported data in our work with cardiomyocytes [11]. These concentrations were found beneficial for culture of cardiomyocytes compared to the peptide-free control hydrogels. Since our ultimate goal is to create a cell-delivery vehicle that would promote cardiomyocyte survival and vascular tube formation, we investigated the same peptide-hydrogel compositions with endothelial cells.

Here we showed that the peptide increased the viability of HUVECs at both the 100 µM and 650 µM concentrations in comparison to the control hydrogel. These studies were performed using monolayers of endothelial cells on peptide modified collagen-chitosan hydrogels (**Figure 4** **–** **6**) to ensure that we observed the effect of the peptide irrespective of the effects related to cell encapsulation such as diffusional limitations in oxygen and nutrient supply [3]. As such, these studies enabled us to assess if the peptide covalently immobilized into the hydrogel retained its functionality in promoting cell survival. To ensure that this statistically significant difference in viability (**Figure 4**) between the peptide modified and non-peptide modified hydrogels was due to the protective nature of the peptide and not due to the peptide increasing proliferation or differential adhesion, cell number and area were examined. It was found that the peptide likely did not enhance endothelial cell proliferation as the average number of cells per area was consistent between the three hydrogel groups (**Figure 5**). As previously mentioned, cell area and cell density assays were performed as monolayer studies of ECs on top of the control and peptide modified collagen-chitosan hydrogels. Since ECs were seeded at a high density and quickly established a confluent monolayer, the effects of the peptide, we believe, would be to promote quiescence in the cells and promote survival similarly to what ang1 has been shown to do in this context specific situation of a confluent monolayer of ECs [31], [32].

HUVECs expressed CD31 in all three hydrogel conditions (**Figure 5**). CD31 staining was performed to ensure that we had an appropriate endothelial cell phenotype on our biomaterial at the various QHREDGS concentrations. If CD31 staining was absent, this would have indicated the hydrogel formulation was not supportive for culture of endothelial cells and expression of their phenotypic markers. CD31 has been shown to be involved in a plethora of endothelial cell functions including: maintenance of adherens junction integrity and permeability, organization of the intermediate filament cytoskeleton, regulation of catenin localization and transcriptional activities, participation in STAT isoform signalling and control of apoptotic events [42], [43].

However, in the 650 µM peptide group we saw that the junction between HUVECs was stronger and less fenestrated in comparison to the 100µM and control hydrogel. This dose dependent relationship the peptide had on creating a less permeable layer of endothelial cells was also seen during 2D tissue culture plastic culture (**Figure 1**). It had been previously shown that ang1 requires the IQ domain GTPase-activating protein 1 as an activator of Rac 1 to regulate barrier function and that this signalling cascade is initiated through the interaction of ang1 and the Tie2 receptor [15], [44].

Since the viability test combined with the cell number analysis pointed towards the peptide’s role in cell survival as opposed to cell proliferation, the protective effect that the peptide had on endothelial cells stressed with paclitaxel was examined. We have shown that there was no significant difference between the taxol treated 100 µM peptide group and the basal levels of caspase-3/7 activation found in the PBS and DMSO controls at this peptide concentration (**Figure 6**). Furthermore, we demonstrated a statistically significant difference in the control and 650 µM peptide groups, between the taxol treated cells and their respective PBS controls (**Figure 6**). Since the most pronounced effect was observed at 100 µM, there was no classical dose-dependency but rather a maximum effect was observed at an intermediate concentration. This kind of behaviour could parallel some of the effects observed in previous studies with ang1. Cascone *et al*. showed enhanced Tie2 activation and signalling at low ang1 concentrations through fibronectin sensitization [18]. They demonstrated that ECs had a 4.4 fold increase in Tie2 phosphorylation with 20 ng/ml of ang1 as compared to only a 2.2 fold increase in Tie2 phosphorylation with 100 ng/ml of ang1 in the presence of a fibronectin based ECM [18]. They also demonstrated that ang1 increased cell adhesion, independent of ECM concentration (collagen, fibronectin or fibrinogen), with an intermediate ang1 concentration being optimal [18]. These findings correlate well with our findings of the 100 µM peptide concentration exhibiting optimal effects in both the caspase-3/7 assay and the encapsulation experiments examining tube formation and cell kinetics.

Ang1 has been found in previous studies to help improve cell survival by preventing apoptosis of endothelial cells when they are under chemical stress or serum deprived conditions [6]. In cardiomyocytes, the protective effect of ang1 was mediated by Akt phosphorylation [9]. The anti-apoptotic effect of ang1 was also found to be dose dependent in endothelial cells and was further enhanced in the presence of VEGF [45]. In our study, we used EGM2 media which contained 2% serum along with VEGF and other endothelial growth factors. Our results, therefore, show a combined protective effect that the peptide had at the lower concentration along with VEGF and serum present in the media. For confluent ECs, which is how our caspase assay was set-up, ang1 was reported in previous studies to induce Tie2 translocation to cell-cell contacts which resulted in the formation of homotypic Tie2-Tie2 trans-associated complexes [31], [32]. These complexes were reported to then preferentially activate PI3K/Akt [46], which resulted in the up-regulation of a broad spectrum apoptosis inhibitor survivin [33].

To determine the behaviour of HUVECs cultured in a 3D environment with the peptide, HUVECs were encapsulated within the three hydrogel groups (**Figure 7** **–** **10**). We found that the peptide modified hydrogels had an increased number of tube-like structures and form these structures at a faster rate in comparison to the control hydrogel with the optimal concentration and time point being 100 µM and 4 days. Ang1 has been shown to promote HUVEC migration in the absence of any other ECM proteins, macromolecules or serum [47]. Furthermore, EC migration has been shown to be Tie2 independent but rather, based on one of the β_1_ integrins [47]. While collagen was present within our hydrogel and could be used to promote migration, the control hydrogel showed a diminished ability to promote cells to organize into tubes.

Tube formation typically involves EC proliferation and sprouting in which tip cells acquire motile behaviour. The extended cellular processes connect by lumen propagation through intercellular and intracellular vacuole fusion [48]. Thus there are many processes involved in tube formation and neovascularization besides cell proliferation [49]. The role ang1 plays in signalling various functionalities in endothelial cells is context dependent based on whether or not ECs are isolated or surrounded by ECs, as previously discussed [31], [32].

In all three encapsulation conditions, regression of the tube-like structures was observed at longer time points. Many primary cells that are cultured *in vitro* cannot be sustained indefinitely. This is due to the lack of a microenvironment that can provide all the necessary mechanical and chemical cues. In this case, the lack of many growth factors [50], absence of supporting cells such as smooth-muscle cells and pericytes [51] and lack of proper matrix stiffness or shear stress [52] for the ECs led to the eventual regression of the tube-like structures.

To further evaluate which integrin this peptide may engage, we performed a two day blocking experiment using integrin antibodies. We chose to block either α5β1 or αvβ3 integrins of encapsulated ECs in the three hydrogel conditions. By blocking integrins α5β1 or αvβ3 for two days immediately after encapsulation, we examined the role that these integrins may play in enabling the cells to attach to the matrix and form tube like structures. Since the QHREDGS peptide is from the fibrinogen-like domain of ang1 [9], we were expecting to see ECs interact with the peptide using the same integrins they use to interact with fibrin. Furthermore, since the matrix we used was collagen, we expected to see the most appreciable changes in tube formation when both collagen and fibrin binding were blocked. We chose to block the integrin receptor αvβ3 which is specific to EC interaction with fibrin to tease out a specific role of the peptide, while not hindering the EC’s ability to interact with collagen [13]. The other integrin receptor blocked was α5β1 since the β1 unit may interact with both collagen and fibrin [13], [53]. Consequently, we expected to see a graded response wherein blocking the integrin receptor α5β1 would more strongly disrupt EC tube formation due to the dual disruption of EC binding to the peptide and collagen matrix while, blocking αvβ3 would only disrupt ECs ability to interact with the peptide while still allowing for ECs to interact freely with the collagen matrix. These integrin receptor blocking experiments have 1) shown that the EC peptide interaction can be inhibited through blocking integrin receptor αvβ3 and that 2) blocking integrin receptor α5β1 resulted in isolated and round ECs after two days of culture possibly due to blocking ECs from interacting with both the peptide and collagen.

We believe that the differences in optimal concentrations for monolayer studies (**Figures 1** **–** **4** **)** and the encapsulation studies (**Figures 7** **–** **10**) are due to the way in which ECs were cultivated and different ways in which the peptide was presented to the cells. Specifically, **Figures 1** **–** **3**, deal with ECs that were cultured in a monolayer and the peptide was applied in the soluble form. When peptide was applied in soluble from, mass transport limitations could be present which would effectively lower the peptide concentration in the vicinity of the cell. When cells were cultivated encapsulated in a hydrogel and the peptide was covalently immobilized to the hydrogel material, the cells were surrounded by the matrix and peptide which allowed for direct and sustained interactions. Too high of a peptide concentration (e.g. 650 µM) could induce cells to adhere too strongly, limiting their ability to migrate and form tubes (as observed in **Figure 8**), thus intermediate peptide concentrations may be most beneficial in terms of the readouts we assessed in this paper. Similar effects were reported previously for other peptides [54], [55]. Our previous work [11], focused on cardiomyocytes, found the optimal concentrations to be 650 µM thus, the differences in the optimal peptide concentrations may be cell type specific (cardiomyocytes vs. endothelial cells).

This study showed that in a dose dependent manner, the peptide QHREDGS can increase cell survival, decrease cell permeability, and induce the formation of tube like structures. We used several different endothelial cell lines (HMVEC-D, MS1 EC, HUVEC**)** in order to confirm that the peptide acted on different endothelial cell types, not just one type, in order to make sure the peptide had a wide applicability. Furthermore, in conjunction with previous studies of QHREDGS and its ability to improve cardiomyocyte survival and function, this peptide has now been shown to have a dual protective nature on endothelial cells and cardiomyocytes. When conjugated to hydrogels, the peptide’s function would be to help ensure initial survival of cells through mitigation of cell apoptosis while stimulating the formation of a primitive vasculature to rapidly form during the first few days to provide oxygen and nutrients to the cells in the hydrogel. Further studies are required to examine the functionality of a combined endothelial cell and cardiomyocyte therapy for myocardial infarction using a QHREDGS modified collagen-chitosan hydrogel *in vivo*.

## Conclusion

We concluded from these experiments that the angiopoietin-1 based peptide QHREDGS was able to promote endothelial cell connectivity, metabolism and survival of endothelial cells cultured in monolayers with peptide applied in soluble form or when covalently immobilized in collagen-chitosan hydrogels. When endothelial cells were encapsulated within a peptide modified collagen-chitosan hydrogel, the peptide was able to promote a more rapid and robust response to form tube-like structures in comparison to the control hydrogel. We propose that the peptide QHREDGS would be an attractive candidate to modify solutions and biomaterials for endothelial cell applications, in order to promote cell survival and migration of cells leading to the formation of new vasculature.
